# Association of De Ritis ratio with oncological outcomes in patients with non-muscle invasive bladder cancer (NMIBC)

**DOI:** 10.1007/s00345-020-03384-9

**Published:** 2020-08-17

**Authors:** Ekaterina Laukhtina, Hadi Mostafaei, David D’Andrea, Benjamin Pradere, Fahad Quhal, Keiichiro Mori, Noriyoshi Miura, Victor M. Schuettfort, Reza Sari Motlagh, Abdulmajeed Aydh, Mohammad Abufaraj, Pierre I. Karakiewicz, Dmitry Enikeev, Shoji Kimura, Shahrokh F. Shariat

**Affiliations:** 1 Department of Urology, Comprehensive Cancer Center, Vienna General Hospital Medical University of Vienna, Währinger Gürtel 18-20, 1090 Vienna, Austria; 2 Institute for Urology and Reproductive Health, Sechenov University, Moscow, Russia; 3 Research Center for Evidence Based Medicine, Tabriz University of Medical Sciences, Tabriz, Iran; 4 Department of Urology, University Hospital of Tours, Tours, France; 5 Department of Urology, King Fahad Specialist Hospital, Dammam, Saudi Arabia; 6 Department of Urology, The Jikei University School of Medicine, Tokyo, Japan; 7 Department of Urology, Ehime University Graduate School of Medicine, Ehime, Japan; 8 Department of Urology, University Medical Center Hamburg-Eppendorf, Hamburg, Germany; 9 King Faisal Medical City, Abha, Saudi Arabia; 10 Division of Urology, Department of Special Surgery, Jordan University Hospital, The University of Jordan, Amman, Jordan; 11 The National Center for Diabetes, Endocrinology and Genetics, The University of Jordan, Amman, Jordan; 12 Cancer Prognostics and Health Outcomes Unit, University of Montreal Health Centre, Montreal, Canada; 13 Department of Urology, Weill Cornell Medical College, New York, NY USA; 14 Department of Urology, University of Texas Southwestern, Dallas, TX USA; 15 Department of Urology, Second Faculty of Medicine, Charles University, Prague, Czech Republic; 16 Karl Landsteiner Institute of Urology and Andrology, Vienna, Austria; 17 European Association of Urology Research Foundation, Arnhem, The Netherlands

**Keywords:** NMIBC, Bladder cancer, RFS, PFS, De Ritis ratio

## Abstract

**Purpose:**

The De Ritis ratio (aspartate aminotransferase/alanine aminotransferase, DRR) has been linked to oncological outcomes in several cancers. We aimed to assess the association of DRR with recurrence-free survival (RFS) and progression-free survival (PFS) in patients with non-muscle-invasive bladder cancer (NMIBC).

**Methods:**

We conducted a retrospective analysis of 1117 patients diagnosed with NMIBC originating from an established multicenter database. To define the optimal pretreatment DRR cut‐off value, we determined a value of 1.2 as having a maximum Youden index value. The overall population was therefore divided into two De Ritis ratio groups using this cut‐off (lower, < 1.2 vs. higher, ≥ 1.2). Univariable and multivariable Cox regression analyses were used to investigate the association of DRR with RFS and PFS. The discrimination of the model was evaluated with the Harrel’s concordance index (C-index).

**Results:**

Overall, 405 (36%) patients had a DRR ≥ 1.2. On univariable Cox regression analysis, DRR was significantly associated with RFS (HR: 1.23, 95% CI 1.02–1.47, *p* = 0.03), but not with PFS (HR: 0.96, 95% CI 0.65–1.44, *p* = 0.9). On multivariable Cox regression analysis, which adjusted for the effect of established clinicopathologic features, DRR ≥ 1.2 remained significantly associated with worse RFS (HR:1.21, 95% CI 1.00–1.46, *p* = 0.04). The addition of DRR only minimally improved the discrimination of a base model that included established clinicopathologic features (C-index = 0.683 vs. C-index = 0.681). On DCA the inclusion of DRR did not improve the net-benefit of the prognostic model.

**Conclusion:**

Despite the statistically significant association of the DRR with RFS in patients with NMIBC, it does not seem to add any prognostic or clinical benefit beyond that of currently available clinical factors.

## Introduction

Approximately 75% of patients with newly diagnosed bladder cancer present with non-muscle-invasive bladder cancer (NMIBC) in developed countries [1]. Despite complete resection and adjuvant intravesical instillation therapy, about 70% of these patients will experience disease recurrence and 30%, eventually, experience progression [2]. Identification of patients who are at high risk of these events would help guide clinical decision making regarding adjuvant treatment indication and regimen as well as intensification of therapy for those at extremely high risk of disease progression, such as early radical cystectomy [3, 4]. Several prognostic models and biomarkers have been investigated [5–8]. However, none of them have been accepted for diagnosis or follow-up in routine practice or clinical guidelines [1, 2].

The ratio of the serum activities of Aspartate Aminotransferase (AST) and Alanine Aminotransferase (ALT), also known as the De Ritis ratio (DRR), was originally proposed as an indicator of liver function damage [9]. Recently, serum levels of DRR have been shown to be associated with outcomes in several urological malignancies [10–13].

However, the literature provides no evidence, to our knowledge, about the value of DRR for prognostication of oncologic outcomes in patients with NMIBC. To fill this gap, we investigated the association of preoperative serum DRR with recurrence-free survival (RFS) and progression-free survival (PFS) in patients treated with transurethral resection of the bladder (TURB) with or without adjuvant intravesical therapy for NMIBC.

## Material and methods

### Study design

We reviewed our established international multicenter database to identify patients treated with TURB for primary or recurrent NMIBC between 1996 and 2007 at four referral centers. We excluded patients with any concomitant malignancy, pelvic radiation; however, present hematological disorders and chronic liver disease within the last 12 months were not excluded*.* Concomitant upper urinary tract carcinoma was excluded in patients with high-risk features with CT or MR urography. Overall, 1117 were available for analysis.

All institutions shared the data agreement contracts before the initiation of the study and provided the necessary clinical data.

### Management and follow-up

All patients underwent a planned complete TURB. A second-look resection was performed 2–6 weeks after initial treatment based on the pathologic and intraoperative findings according to guidelines at the time [1, 14]. A second look was indicated in case of incomplete initial TURB or in case of doubt about completeness of a TURB; if there is no muscle in the specimen after initial resection, with the exception of TaLG/G1 tumors and primary CIS; in T1 tumors. Immediate and/or adjuvant intravesical therapy was administered at the discretion of the treating physician and according to guidelines at the time.

All surgical resection specimens were processed according to standard pathologic procedures by dedicated genitourinary pathologists at each participating institution. The pathologic stage was reassigned using the 2010 American Joint Committee on Cancer TNM staging system and tumor grade according to the 1973 World Health Organization (WHO) grading system. All specimens were re-evaluated by a dedicated uropathologist. Based on pathological T stage, pathological grade, concomitant CIS, prior recurrence rate (primary vs. ≤ 1 recurrence/year vs. > 1 recurrence/year), tumor diameter (< 3 cm vs. ≥ 3 cm) and focality (single vs. 2–7 vs. ≥ 8), patients were stratified in low, intermediate and high risk groups in accordance with 2018 European Association of Urology guidelines as well as European Organization for Research and Treatment of Cancer risk tables (EORTC) [1].

All laboratory tests were done within 30 days before TURB. To define the optimal pretreatment DRR cut‐off value, we carried out a time‐dependent receiver operating characteristic curve analysis for 3‐year RFS as the end‐point, considering the median RFS time (12 months), and determined a value of 1.2 as having a maximum Youden index value. The overall population was therefore divided into two De Ritis ratio groups using this cut-off (lower < 1.2 vs. higher ≥ 1.2).

Due to the retrospective nature of the study, there was no standardized follow-up. In general, follow-up was performed in accordance with institutional protocols and guidelines at the time. It usually included urinary cytology and a cystoscopy every 3 months for the first 2 years after surgery; after that, every 6 months for 3 years, and then, annually. Patients with suspected disease recurrence underwent a repeated TURB. Disease recurrence was defined as the first tumor relapse in the bladder regardless of tumor stage. Disease progression was defined as tumor relapse at tumor stage T2 or higher.

### Statistical analysis

Univariable and multivariable Cox regression analyses were used to evaluate the association of DRR with RFS and PFS. Kaplan–Meier survival curves were used to graphically visualize the correlation between DRR and the time to recurrence and progression. The log-rank test was used to determinate the statistical difference between the DRR < 1.2 and DRR ≥ 1.2 groups with respect to disease recurrence and progression. The discrimination of the model was evaluated using the Harrel’s concordance index (C-index). Decision curve analysis (DCA) was used to assess the clinical impact on decision making of preoperative DRR. In brief, the method of DCA is based on the principle that the relative harms of false positives and false negatives can be expressed in terms of a probability threshold. This threshold probability can be used, both, to determine whether an individual patient's test result should be defined as positive or negative and to weight the clinical consequences of true and false. The decision analytic evaluation should be performed during later stages of research before clinical implementation of the biomarker. Statistical significance was set at *p* < 0.05. All tests were 2-sided. Analyses were performed using STATA, version 16.0 (StataCorp LP, College Station, TX).

## Results

Among 1117 NMIBC patients with median (IQR) age 67 (58–74) years, 931 patients (83.3%) had primary tumor and 718 (64.3%)—single tumor. 653 (58.5%) patient had pTa stage tumor, 21 (1.9%)—pTis, and 443 (39.6%)—pT1. Overall, 405 (36%) patients had a DRR ≥ 1.2 and 712 (64%) a DRR < 1.2. There was no difference in clinicopathologic features between two groups (Table 1). High DRR levels were correlated with more common use of intravesical BCG treatment (*p* < 0.05).

**Table 1:** Clinicopathologic features of 1117 patients treated with transurethral resection of the bladder (TURB) for NMIBC, stratified by the De Ritis ratio (DRR)

Parameters	All	DRR < 1.2	DRR ≥ 1.2	*p* value
Total, *n* (%)	1 117	712 (63.7)	405 (36.3)	
Age, median (IQR)	67 (58–74)	66 (58–74)	67 (59–74)	0.4
Female gender, *n* (%)	262 (23.5)	178 (25.0)	84 (20.7)	0.1
Tumor stage, *n* (%)				0.7
pTa	653 (58.5)	413 (58.0)	240 (59.3)	
pTis	21 (1.9)	12 (1.7)	9 (2.2)	
pT1	443 (39.6)	287 (40.3)	156 (38.5)	
Tumor grade, *n* (%)				0.7
G1	231 (20.7)	151 (21.2)	80 (19.7)	
G2	398 (35.6)	247 (34.7)	151 (37.3)	
G3	488 (43.7)	314 (44.1)	174 (43.0)	
Concomitant carcinoma in situ, *n* (%)	66 (5.9)	41 (5.8)	25 (6.2)	0.8
Tumor size, *n* (%)				0.6
< 1 cm	368 (32.9)	242 (34.0)	126 (31.1)	
1–3 cm	448 (40.1)	284 (40.0)	164 (40.5)	
> 3 cm	301 (27.0)	186 (26.0)	115 (28.4)	
Number of tumors, *n* (%)				0.2
1 tumor	718 (64.3)	469 (65.9)	249 (61.5)	
1–7 tumors	297 (26.6)	176 (24.7)	121 (29.9)	
≥ 8 tumors	102 (9.1)	67 (9.4)	35 (8.6)	
Smoker, *n* (%)				0.1
Never	272 (24.4)	186 (26.1)	86 (21.2)	
Former	331 (29.6)	216 (30.4)	115 (28.4)	
Current	514 (46.0)	310 (43.5)	204 (50.4)	
Intravesical therapy, *n* (%)	493 (44.1)	293 (41.2)	200 (49.4)	0.01
Type of intravesical therapy, *n* (%)				0.001
No intravesical therapy	624 (55.9)	419 (58.8)	205 (50.6)	
Adjuvant BCG	300 (26.9)	158 (22.2)	142 (35.1)	
Adjuvant chemotherapy	48 (4.3)	30 (4.2)	18 (4.4)	
Early single instillation	145 (12.9)	105 (14.8)	40 (9.9)	
Prior recurrence, *n* (%)				0.1
Primary tumor	931 (83.3)	584 (82.0)	347 (85.7)	
Recurrent tumor	186 (16.7)	128 (18.0)	58 (14.3)	
EORTC risk for recurrence, *n* (%)				0.9
Low	443 (39.7)	283 (39.7)	160 (39.5)	
Intermediate	524 (46.9)	333 (46.8)	191 (47.2)	
High	150 (13.4)	96 (13.5)	54 (13.3)	
EORTC risk for progression, *n* (%)				0.6
Low	558 (49.9)	350 (49.2)	208 (51.4)	
Intermediate	432 (38.7)	283 (39.7)	149 (36.8)	
High	127 (11.4)	79 (11.1)	48 (11.8)	

Statistical significance was set at *p* < 0.05

**Fig. 1:**
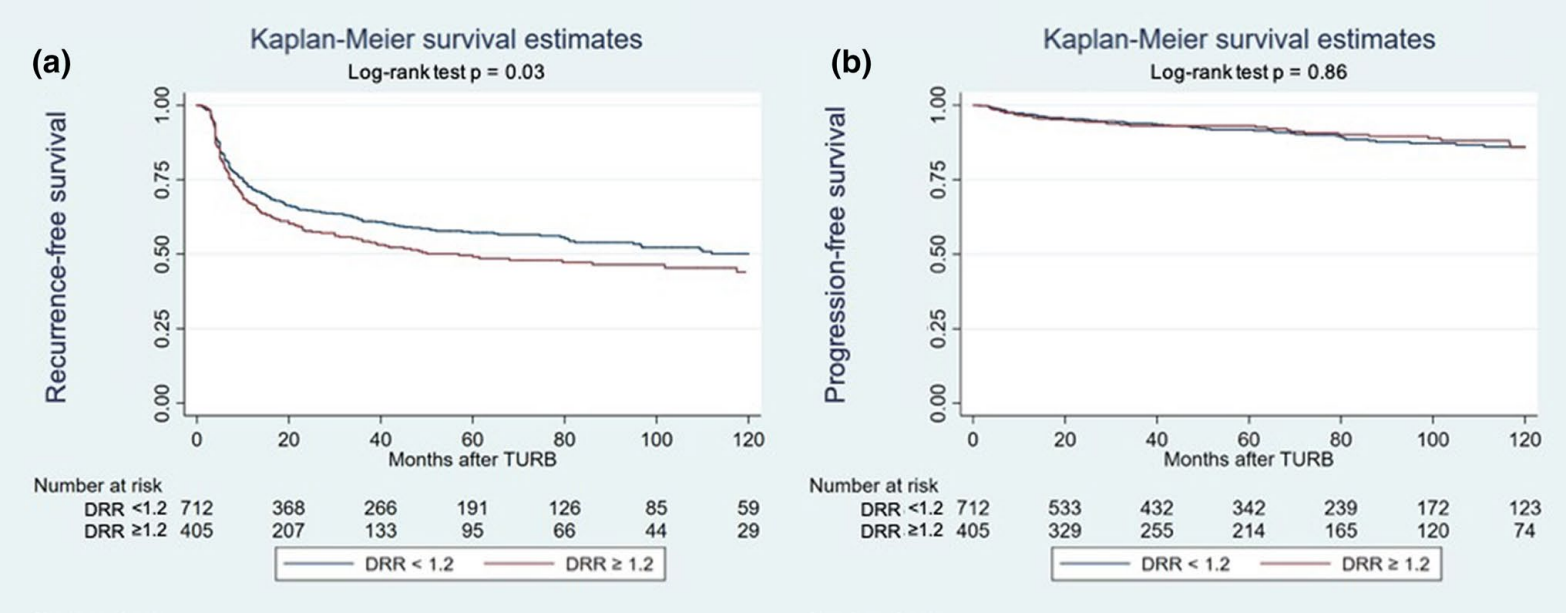
Kaplan–Meier analysis for recurrence-free survival (**a**) and progression-free survival (**b**) in 1117 patients treated with transurethral resection of the bladder (TURB) for non-muscle-invasive bladder cancer, stratified by De Ritis ratio (DRR) at a cut-off of 1.2

Within a median follow-up of 64 (IQR: 26–100) months, a total of 469 (42.0%) patients experienced disease recurrence and 103 (9.2%) patients—disease progression. The correlation of DRR with RFS and PFS was graphically estimated using Kaplan–Meier survival curves (Fig. 1).

On univariable Cox regression analyses, preoperative serum DRR was associated with RFS (HR: 1.23, 95% CI 1.02–1.47, *p* = 0.03) but not with PFS (HR: 0.96, 95% CI 0.65–1.44, *p* = 0.9) (Table 2).

**Table 2:** Univariable and multivariable Cox regression analysis predicting recurrence-free survival (RFS) and progression-free survival (PFS) in patients with NMIBC

Variables	RFS				PFS			
	Univariable		Multivariable		Univariable		Multivariable	
	HR (95% CI)	*p* value	HR (95% CI)	*p* value	HR (95% CI)	*p* value	HR (95% CI)	*p* value
Gender (female)	1.04	0.7	0.96	0.7	1.15	0.5	1.09	0.7
Age	1.02	< 0.001	1.02	< 0.001	1.04	< 0.001	1.04	< 0.001
De Ritis	1.23	0.03	1.21	0.04	0.96	0.9	0.91	0.7
Number of tumors								
1 tumor	Ref	Ref	Ref	Ref	Ref	Ref	Ref	Ref
1–7 tumors	1.51	< 0.001	1.48	< 0.001	0.43	0.1	1.13	0.6
≥ 8 tumors	1.04	0.8	1.09	0.6	2.54	0.001	2.11	0.01
Tumor size								
< 1 cm 1–3 cm	Ref 0.98	Ref 0.9	Ref 1.06	Ref 0.6	Ref 0.45	Ref 0.1	Ref 1.39	Ref 0.2
> 3 cm	2.42	< 0.001	2.3	< 0.001	1.71	0.04	1.41	0.2
Tumor stage								
pTa	Ref	Ref	Ref	Ref	Ref	Ref	Ref	Ref
pTis	0.78	0.5	1.02	0.9	1.79	0.4	1.36	0.7
pT1	0.71	< 0.001	0.41	< 0.001	1.57	0.02	0.43	0.02
Tumor grade								
G1	Ref	Ref	Ref	Ref	Ref	Ref	Ref	Ref
G2	1.99	< 0.001	1.59	0.001	2.52	0.02	2.17	0.05
G3	1.27	0.1	2.59	< 0.001	4.01	< 0.001	7.12	< 0.001
Concomitant CIS	0.95	0.8	0.92	0.7	1.43	0.4	0.91	0.8
Intravesical therapy	0.58	< 0.001	0.55	< 0.001	1.14	0.5	0.93	0.8
C-index with Deritis	0.683				0.713			
C-index without Deritis	0.681				0.713			

Statistical significance was set at *p* < 0.05

**Fig. 2:**
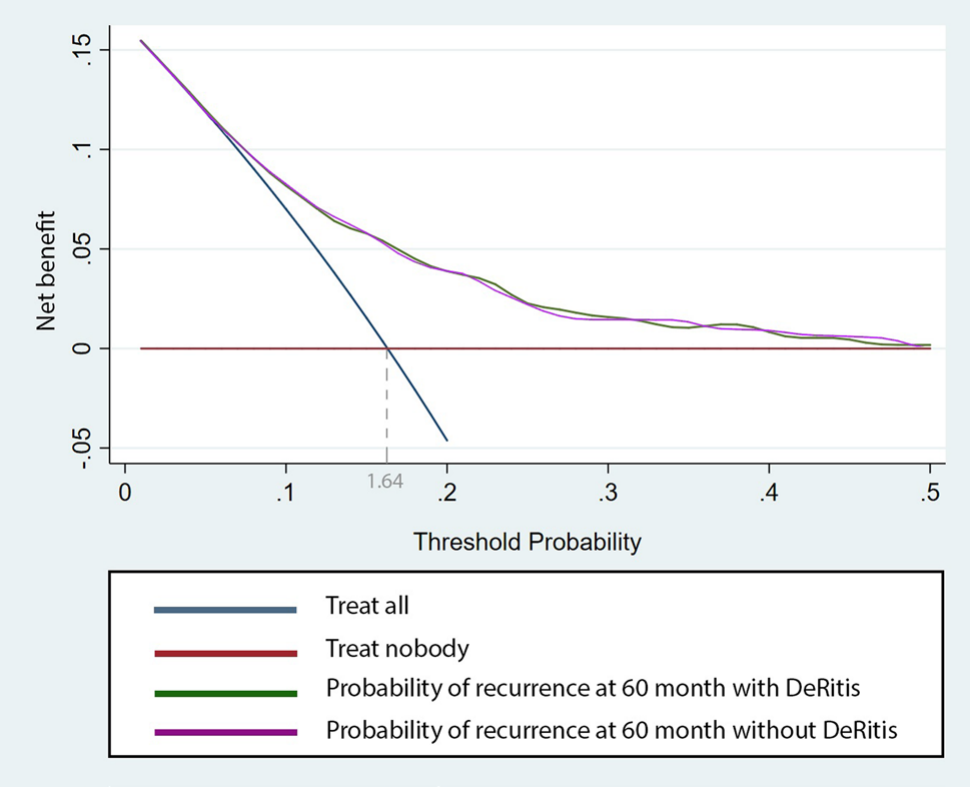
Decision curve analysis assessing the clinical impact of current prognostic models (Base model) with the integration of the De Ritis ratio (DRR model) estimating probability of recurrence at 60 months, in 1117 patients treated with transurethral resection of the bladder (TURB) for NMIBC. The two models are compared with the strategies of treating all or none of the patients with TURB

On multivariable Cox regression analysis which adjusted for the effects of age, gender, stage, concomitant CIS, tumor size and the administration of adjuvant intravesical therapy, preoperative serum DRR ≥ 1.2 remained associated with worse RFS (HR: 1.21, 95% CI 1.00–1.46, *p* = 0.04). The addition of the DRR to a multivariable base model, that includes all established predictors, improved its discrimination only negligible (C-index = 0.683 vs. C-index = 0.681) (Table 2). On DCA, the base model including pathological T stage, pathological grade, concomitant CIS, prior recurrence rate, tumor diameter, and focality added a value to any clinical decision making at a threshold probability between 6 and 50%. The inclusion of the DRR did not improve the net-benefit of the model (Fig. 2).

**Fig. 3:**
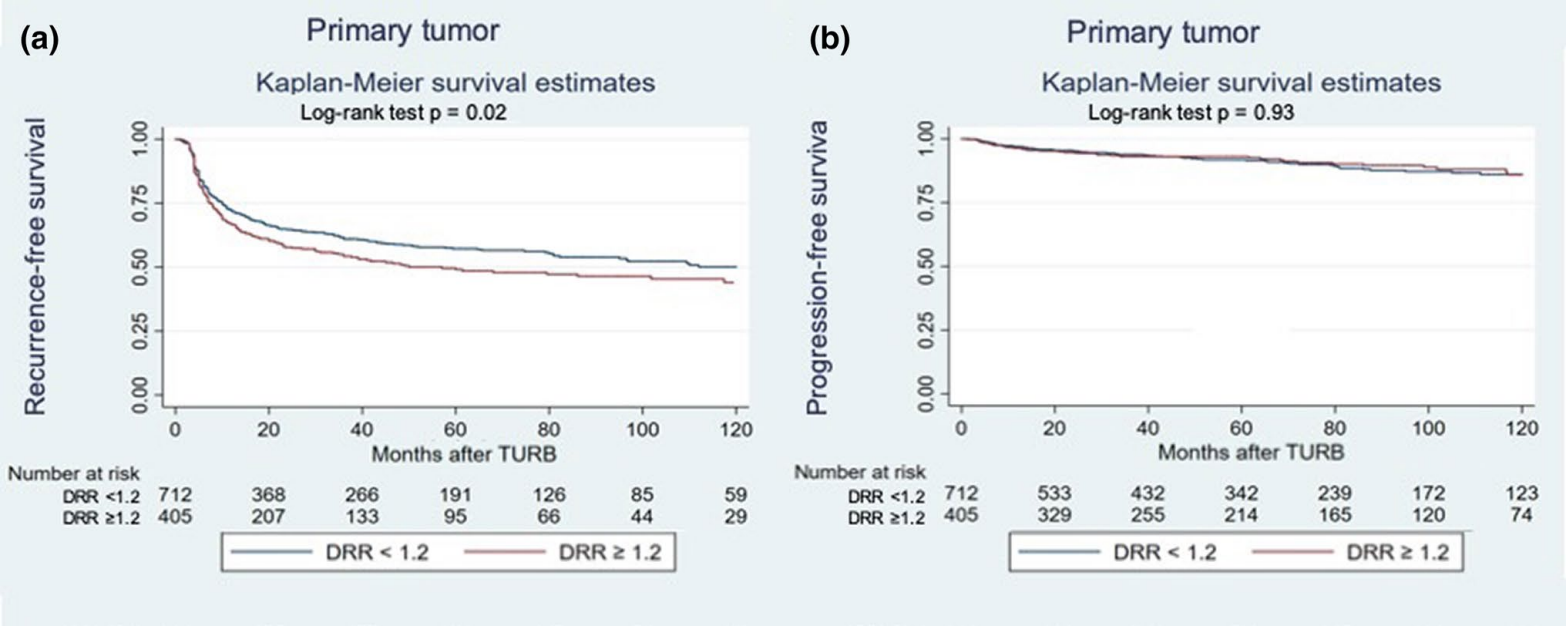
Recurrence-free survival (**a**) and progression-free survival (**b**) estimates for patients treated with transurethral resection of the bladder (TURB) for primary NMIBC, stratified by De Ritis ratio (DRR) cut-off of 1.2

In patients with primary NMIBC, preoperative serum DRR ≥ 1.2 was associated with worse RFS (HR: 1.25, 95%CI 1.02–1.52, *p* = 0.02, Fig. 3). In patients with recurrent NMIBC, preoperative serum DRR was associated with neither RFS nor PFS (all *p* > 0.05). On exploratory subgroup analyses based on the type of adjuvant instillation therapy administered, DRR was still not associated with RFS or PFS (all *p* > 0.05).

## Discussion

The exact mechanism of interaction of DRR with cancer and its increase with tumor aggressiveness is still to be uncovered. One hypothesis is that the link is through aerobic glycolysis, which was shown to occur in actively proliferating cancer cells through the employment of AST rather than that of ALT [15, 16]. This would suggest that the increased DRR indicates the generation of increased oxidative stress [17].

We investigated the association of the preoperative serum DRR with oncologic outcomes in NMIBC patients. We found preoperative serum DRR ≥ 1.2 to have an independent association with worse RFS. These findings are in agreement with a previous retrospective analysis of 118 patients treated with radical cystectomy for non-metastatic urothelial BCa [18]. In that study, on multivariable Cox regression analysis, a high DRR, defined as ≥ 1.3, was independently associated with metastasis, cancer-related death and overall death. We expanded upon these previous findings by analyzing a large cohort of patients with NMIBC originating from a multicenter cooperative database. In addition, we evaluated the C-index and DCA. Biomarkers should provide unique information that adds to known clinical and pathologic information [19]. Conventional multivariable analyses are not sufficient to demonstrate improved prediction of outcomes. Predictive models, including or excluding any new putative biomarker, need to show clinically significant improvement of performance in order to claim any real benefit. We found that preoperative serum DRR does not add any prognostic information beyond that afforded by standard clinical factors. Indeed, the change in C-index by adding serum preoperative DDR to these factors was marginal and negligible. This measure (i.e., C-index) quantifies the ability of the model to discriminate between patients with and those without the outcome of interest [20]. In addition, for a biomarker to have clinical value, it needs to change the clinical consequences. This type of analysis allows insight into the consequences of using a biomarker in the clinic [21]. We used the decision curve analysis which is a method that combines simplicity with efficient computations [22]. Unfortunately, here as well, preoperative serum DDR did not show any net clinical benefit over the established clinical factors, regardless of the probability threshold used.

However, we did not find any association of DRR with PFS in NMIBC, both on univariable and multivariable Cox regression analyses. In contrast, several studies have reported a relationship between DRR and the progression of various other urological cancers such as renal cell [23], prostate [12, 24] and testicular [13] cancers. This could be due to the different disease and the severity of disease state as well as the patient population in general. In addition, in our study, high preoperative DRR associated with worse RFS in patients with primary tumor. However, we failed to find an association with RFS or PFS in patients with recurrent NMIBC. We suppose that these results may be explained by the predominant number of patients we had in the primary tumor group (*n* = 931).

It is also important to highlight that combining DRR with other biomarkers may improve the accuracy of the prognosis model. Several other prognostic tissue and serum-based biomarkers have been investigated [5, 25]. For instance, neutrophil-to-lymphocyte ratio (NLR) was found significantly associated with both RFS and PFS in primary NMIBC patients [26]. Although we did not find an association between DRR and PFS. It should be noticed that the studies in this field employed different biomarkers cut-offs. Another marker of systemic inflammation, C-reactive protein, was also associated with a higher risk of disease recurrence and progression in NMIBC [27]. Among the potential confounders, there were a relatively low rate of intravesical instillations and missed data on prior TURB and re-TURB. Another study showed that serum cholinesterase was significantly associated with shorter RFS in patients with NMIBC undergoing TURB [28]. In our study, DRR was also shown to be associated only with RFS. However, most of the aforementioned studies were limited at their retrospective and multicenter designs limiting them to hypothesis-generating status. Indeed, the limitations in study design and the overall lack in adherence to a structured biomarker testing and validation process have impaired the progress of biomarkers in NMIBC to clinical practice [19, 29].

Our study is not devoid of limitations, which are mainly inherent to its retrospective design. First, we could not control for surgical quality and data on second-look TURB were not available. Second, the administration of adjuvant intravesical therapy was not standardized. Third, confounding diseases such as known hematological disorder, chronic liver disease, the presence of undetected liver disease or drug interaction might affect the DRR, thereby leading to false-negative findings. Despite all these limitations, we present the largest series investigating the association of DRR with oncologic outcomes in NMIBC.

## Conclusion

Despite the statistically significant association of the DRR with RFS in patients with NMIBC, it does not seem to add any additive value to current prognostic models in NMIBC. Further studies could investigate its association with response to adjuvant therapies such as intravesical and systemic immunotherapies.
